# MRI in acute muscle tears in athletes: can quantitative T2 and DTI predict return to play better than visual assessment?

**DOI:** 10.1007/s00330-020-06999-z

**Published:** 2020-07-15

**Authors:** J. D. Biglands, A. J. Grainger, P. Robinson, S. F. Tanner, A. L. Tan, T. Feiweier, R. Evans, P. Emery, P. O’Connor

**Affiliations:** 1 NIHR Leeds Biomedical Research Centre, Leeds Teaching Hospitals NHS Trust, Leeds, UK; 2 Medical Physics and Engineering, Leeds Teaching Hospitals NHS Trust, Leeds, UK; 3 Leeds Institute of Rheumatic and Musculoskeletal Medicine, Chapel Allerton Hospital, University of Leeds, Leeds, UK; 4 Siemens Healthcare GmbH, Erlangen, Germany

**Keywords:** Skeletal muscles, Diffusion tensor MRI, Multi-parametric MRI, Muscle tear

## Abstract

**Objectives:**

To assess the ability of quantitative T2, diffusion tensor imaging (DTI) and radiologist’s scores to detect muscle changes following acute muscle tear in soccer and rugby players. To assess the ability of these parameters to predict return to play times.

**Methods:**

In this prospective, longitudinal study, 13 male athletes (age 19 to 34 years; mean 25 years) underwent MRI within 1 week of suffering acute muscle tear. Imaging included measurements of T2 and DTI parameters. Images were also assessed using modified Peetrons and British athletics muscle injury classification (BAMIC) scores. Participants returned for a second scan within 1 week of being determined fit to return to play. MRI measurements were compared between visits. Pearson’s correlation between visit 1 measurements and return to play times was assessed.

**Results:**

There were significant differences between visits in BAMIC scores (*Z* = − 2.088; *p* = 0.037), modified Peetrons (*Z* = − 2.530; *p* = 0.011) and quantitative MRI measurements; T2, 13.12 ms (95% CI, 4.82 ms, 21.42 ms; *p* = 0.01); mean diffusivity (0.22 (0.04, 0.39); *p* = 0.02) and fractional anisotropy (0.07 (0.01, 0.14); *p* = 0.03). BAMIC scores showed a significant correlation with return to play time (*R*_s_ = 0.64; *p* = 0.02), but modified Peetrons scores and quantitative parameters did not.

**Conclusions:**

T2 and DTI measurements in muscle can detect changes due to healing following muscle tear. Although BAMIC scores correlated well with return to play times, in this small study, quantitative MRI values did not, suggesting that T2 and DTI measurements are inferior predictors of return to play time compared with visual scoring.

**Key Points:**

• *Muscle changes following acute muscle tear can be measured using T2 and diffusion measurements on MRI*.

• *Measurements of T2 and diffusion using MRI are not as good as a radiologist’s visual report at predicting return to play time after acute muscle tear*.

## Introduction

Acute muscle tears are common in athletes and have a particularly high prevalence in the lower limb [1]. These injuries are responsible for a significant loss of time spent in competition, with pressure on medical teams to return athletes to competition rapidly [2]. However, premature return may result in recurrent injury and a longer period of convalescence [3]. Subsequently, injury diagnosis and prediction of time to return to play are active areas of research, including injury classification using magnetic resonance imaging (MRI) [2–5].

The modified Peetrons [6, 7] and British athletics muscle injury classification (BAMIC) [8, 9] scores are the most commonly used MRI muscle injury grading techniques. They are based on the site and size of injury within the muscle and the morphological features of the tear. Numerous studies have investigated the ability of standard MRI to predict return to play times, with the majority finding no association [6, 10–16]. However, the ability of quantitative MRI to predict return to play times has not been assessed.

Quantitative MRI measurements may be useful as an objective assessment of muscle tears. T2 measurements have been shown to be able to detect subtle muscle changes due to disease [17, 18] and exercise [19, 20] and can also show muscle degeneration and regeneration after femoral artery ligation in mice [21]. DTI parameters also have potential as a quantitative assessment of muscle, being particularly responsive to the size and directionality of water diffusivity in injured muscle [22]. STEAM-DTI measurements have been shown to be able to detect differences between muscle tear and healthy muscle in the same patient [23] and quantitative MRI has been shown to be useful in cross-sectional studies [21–23]. However, the ability of quantitative MRI to detect longitudinal changes due to healing after acute muscle tear has not yet been assessed.

The aims of this longitudinal, observational study were to assess the ability of quantitative T2 and STEAM-DTI parameters to detect muscle changes following acute muscle tear and to assess the correlation between these parameters and return to play times. We tested the hypotheses that there was no difference in quantitative MRI measurements at the time of tear and at time of returning to play, and that there was no correlation between these measurements and return to play time.

## Methods

### Participants

Consecutive referrals, from professional soccer and rugby clubs, for clinically indicated MR examination of a muscle injury were recruited into this study. Participants gave written, informed consent to take part with approval of the National Research Ethics Service (17-EM-0079). The study was conducted according to the Declaration of Helsinki. Sample size was based on published rules of thumb for estimating parameters for powering future clinical trials [24]. Inclusion criteria were clinical evidence of a muscle tear in the lower limb with at least 2 of the following criteria present: history of pain in a muscle group commencing during sporting activity, pain on walking 24 h after injury, local tenderness to palpation of the affected muscle, reduced muscle power and range of movement on specific muscle testing (i.e., 90–90 test for the hamstrings).

Exclusion criteria were contraindications for MRI, re-injury on return to play (which was considered a premature return to play) or non-attendance at the follow-up scan. Participants were not excluded based on age, comorbidities or previous muscle injury. The recruitment period extended from 11 March 2016 to 18 April 2017.

### MR imaging

Initial MR imaging was performed 0–7 days following muscle injury (visit 1). The sports teams were provided with a report of the MR scan, which included a description of the site and size of the muscle injury in keeping with the ethical requirements of the study, but were blinded to tear study scores on MRI. Follow-up imaging was performed 0–7 days after the participant was classed as fit to return to play (visit 2). Criteria for the return to play decision-making included asymptomatic completion of a rehabilitation program and a subjective clinical assessment by the sports medicine team. Return to play time was recorded as the time from injury until the participant returned to full, unrestricted training.

Image acquisition was performed using a previously described protocol [25]. MR data were acquired using a MAGNETOM Verio 3-T MR scanner (Siemens Healthcare) using two, small, four-channel flex coils wrapped around the injured leg. The scan parameters for the imaging are given in Table 1. The fields of view for all imaging sequences were fixed at 300 × 300 mm^2^. The musculoskeletal radiologist identified the slice position of the centre of the muscle tear site on the short tau inversion recovery (STIR) volume (Table 1). The T2 and diffusion images were then aligned with this slice position.

**Table 1:** MRI scan parameters for T1-weighted, T2 measurements and STEAM diffusion scans

	T1 weighted	T2	Diffusion
Imaging sequence	Turbo spin-echo (TSE)	Multi-echo, spin-echo (MESE)	STEAM-EPI
TR: repetition time (ms)	697	1500	6300
TE: echo time(s) (ms)	9.1	9.6:9.4:153.6 (16 echos)	42.2
Field of view (mm)	300 × 300	300 × 300	300 × 300
Slice thickness (mm)	5	5	5
Fat suppression	STIR	SPAIR	SPAIR
Acquisition matrix	256 × 256	256 × 256	256 × 256
Number of slices	60	5	5
Number of averages	1	1	8
Receiver bandwidth (Hz/pixel)	222	510	1502
Flip angle (°)	90	15	-
GRAPPA	-	-	2
*b* values (s/mm^2^)	-	-	0, 500
Directions	-	-	6
Mixing time (Tm) (ms)	-	-	980
Diffusion time (∆) (ms)	-	-	1000
Acquisition time (min:s)	2:19	2:05	6:12

Diffusion-weighted images were acquired using a STEAM prototype sequence. To measure T2, a multi-echo spin-echo (MESE) sequence was used. A volume-interpolated breath-hold examination (VIBE), 2-point Dixon sequence, TR 11 ms, TE 2.45 ms and 3.675 ms, flip angle 15°, duration 94 s, was also acquired. These Dixon images were used to obtain signal-weighted fat-fraction estimates to check region of interest (ROI) placement for subcutaneous and fascial fat.

### Image analysis

Modified Peetrons [7] and BAMIC [9] semi-quantitative muscle tear scoring was performed on anonymised STIR axial image volumes by a consultant musculoskeletal radiologist (P.R.) who was blinded to the visit ordering. In order to assess correlations, BAMIC scores were converted to an ordinal scale. ROIs defining the tear site, oedematous muscle, normal muscle and any haematoma were drawn on a single slice at both visit 1 and visit 2 images by a separate consultant musculoskeletal radiologist (P.J.O.C.). Scoring and ROI drawing were performed by two different radiologists (both with over 20 years of experience of muscle tear imaging) to avoid memory bias from the contouring stage.

**Fig. 1:**
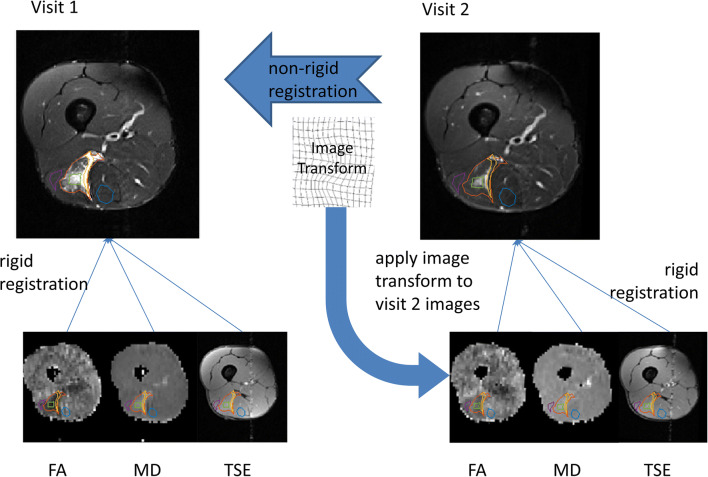
The image registration process. To correct for between-scan movement, the visit 2, T1-weighted, axial STIR image was registered to the corresponding visit 1 image. The resulting affine image transform was then applied to all visit 2 image sets. Subsequently, to correct for within-scan motion, all image datasets in each visit were aligned to the STIR image for that visit using rigid registration

To make direct comparisons between visits, ROIs drawn on the STIR images were aligned between visits using image registration. Movement between different acquisitions within the visit was also corrected for. The image registration process is illustrated in Fig. 1 and is described as follows:

1. An initial bounding box was defined around the leg to exclude the contralateral leg using threshold analysis.
2. For alignment between visits, two-dimensional affine image registration was applied to the STIR images at the tear site slice. The optimisation of the transform parameters was achieved with a regular-step gradient descent optimiser [26] and Mattes’ implementation of the mutual information image similarity metric [27].
3. The image transform obtained in step 2 was then applied to the remaining visit 2 images, i.e. the DTI maps and TSE images.
4. For within-visit alignment, a rigid registration was used. As patient movement within a single scanning session was small, it was expected that rigid registration would be adequate for this step.

Registration was evaluated on a case-by-case basis by two consultant musculoskeletal radiologists (P.J.O.C., A.G.) based on whether the ROIs visually aligned with the tear site on the target visit images. In some cases, haematoma (detected at visit 1 in close proximity to subcutaneous fat) had completely resolved by visit 2, which could result in subcutaneous fat being classified as haematoma in visit 2. Therefore, visit 2 ROIs that had a signal-weighted fat-fraction of over 20% were assumed to contain signal outside the muscle and were excluded from the study.

To generate T2 values, the signal intensity versus echo time decay curves from each ROI were fitted with a mono-exponential function including a constant term to account for the noise floor. The earliest time point in the echo train was excluded from the fit [28]. Mean diffusivity (MD), fractional anisotropy (FA) and diffusion eigenvalue (*λ*_1_, *λ*_2_, *λ*_3_) maps were generated using the scanner vendor’s software.

Two comparative studies to assess healing were made. In the first, quantitative MRI parameters from ROIs drawn at visit 1 were compared between visits. These changes are important to understand because the visit 1 measurements are the basis for predicting return to play time. However, this comparison reflects healing changes both within the tear site and due to reduction of the size of the tear. Therefore, to assess changes within the tear site independent of tear size reduction, a second comparison was performed. Here, quantitative MRI parameters from tear site ROIs drawn in the remaining tear at visit 2 were compared with the values at visit 1 at the same position. Because both ROIs in this comparison lay within the tear site, these measurements were only susceptible to changes within the tear site itself. Finally, the correlation between visit 1 measurements and return to play time was assessed.

### Statistics

Statistical analysis was performed in SPSS (IBM SPSS, version 25.0). Differences between continuous variables are expressed as mean difference (95% confidence interval), *p* value, unless otherwise stated. Differences between continuous variables were compared using a paired *t* test or a Wilcoxon signed-rank test for data parameters that were not normally distributed. Differences between ordinal variables, such as radiologist’s semi-quantitative scores, were assessed using a Wilcoxon signed-rank test (*Z*-score). Correlation was assessed using Spearman’s rank-order correlation for ordinal scores and Pearson’s correlation for continuous measurements.

## Results

### Participants

Twenty-one male athletes with acute muscle tears were recruited into the study, of which thirteen returned for a second scan (mean age 25 years, range 19 to 34 years (8 did not want to take the time to return for a second scan)). Eight tears were situated in the biceps femoris (6 right side, 2 left side), two in the semitendinosus (both left side), 2 in the soleus (1 each side) and 1 in the gastrocnemius (right side). Eleven tears were partial thickness injuries (BAMIC 1–3, modified Peetrons 1–2), and two were complete muscle tears (BAMIC 4, modified Peetrons 3). The mean return to play time was 31 days (range, 17 to 56 days). Players were followed up for 1 year during which none of the recruited athletes had a re-tear at the site of the study injury.

### Registration

Radiologists visually confirmed that the image registration was accurate in all cases. Seven of the visit 2 haematoma ROIs were excluded from the analysis because the haematoma had resolved, leaving the haematoma ROI in fascial or subcutaneous fat (fat fraction > 20%).

### Longitudinal study

There was a significant change in BAMIC score between visits (*Z* = − 2.088, *p* = 0.037). The median BAMIC score was 2b at visit 1 and 1b at visit 2. In 5/13 participants, the BAMIC score did not reduce between visits. The mean differences in quantitative MRI parameters in these cases were T2, 14.2 (95% CI, − 2.1, 30.4) ms; MD, 0.20 (− 0.41, 0.80) × 10^−3^ mm^2^s^−1^; and FA, − 0.08 (− 0.15, − 0.01) for the tear site ROI.

There was a significant change in modified Peetrons (*Z* = − 2.530, *p* = 0.011) between visits. Median Peetrons score stayed at 2 between visits. In 6/13 participants, the Peetrons score did not reduce between visits. The mean differences in quantitative MRI parameters in these cases were T2, 13.9 (95% CI, 3.7, 24.2) ms; MD, 0.18 (− 0.11, 0.47) × 10^−3^ mm^2^s^−1^; and FA, − 0.07 (− 0.03, − 0.11).

### Visit 1 ROIs

**Fig. 2:**
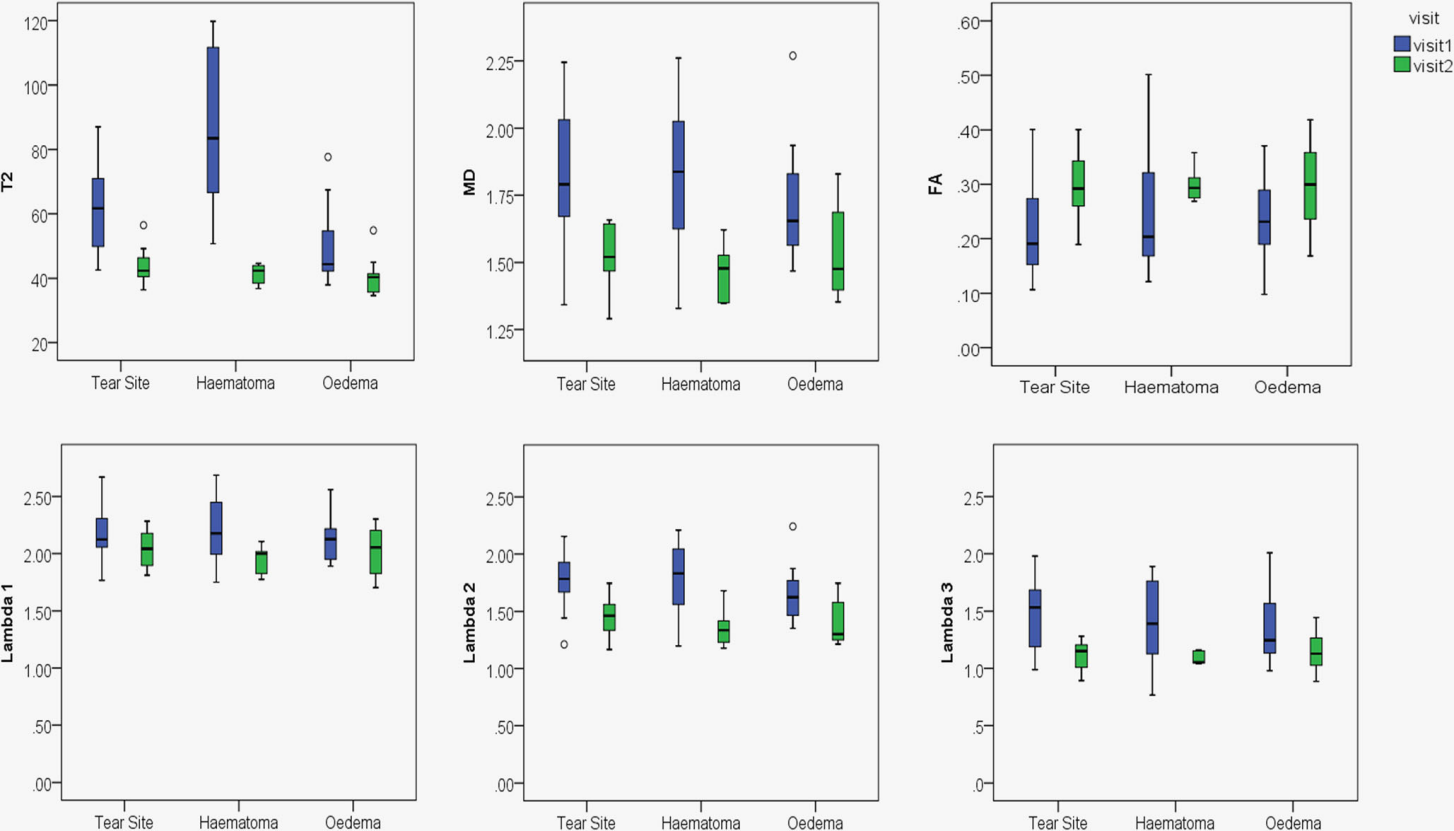
Boxplots for quantitative MR parameters obtained from ROIs drawn on visit 1 and propagated to visit 2. Measurements show tissue changes and the effect of the tear shrinking. Values for T2, MD and FA and eigenvalues in tear site, haematoma and oedema for both visits are presented

Considering the ROIs drawn on the initial (visit 1) images, incorporating the whole of the initial tear, T2 values were significantly lower at visit 2 in the tear site, haematoma and oedema ROIs (Fig. 2, Table 2). MD, *λ*_2_ and *λ*_3_ were significantly reduced after healing in the tear site and oedema, and FA was increased. There was no significant change in *λ*_1_ and there were no significant longitudinal differences in normal muscle for any of the measures.

**Table 2:** Quantitative MR parameters obtained from ROIs drawn on visit 1 and propagated to visit 2. Measurements show tissue changes and the effect of shrinking tear size. Values for T2, MD and FA and eigenvalues in tear site, haematoma and oedema are presented. Mean values for both visits and the difference between them are presented as mean (confidence interval); *p* value

ROI measurement	Visit 1 mean (CI)	Visit 2 mean (CI)	Difference mean (CI); *p* value
Tear site			
T2 (ms)	63.4 (54.2, 72.7)	43.5 (39.9, 47.1)	19.9 (11.5, 28.3); *p* < 0.001
MD (×10^−3^ mm^2^ s^−1^)	1.86 (1.67, 2.04)	1.53 (1.45, 1.62)	0.32 (0.09, 0.55); *p* = 0.001
FA	0.19 (0.15, 0.23)	0.30 (0.26, 0.34)	0.11 (0.17, 0.06); *p* = 0.001
*λ*_1_(×10^−3^mm^2^s^−1^)	2.21 (2.02, 2.04)	2.03 (1.92, 2.14)	0.18 (− 0.05, 0.41); *p* = 0.12
*λ*_2_(×10^−3^mm^2^s^−1^)	1.80 (1.61, 1.99)	1.45 (1.34, 1.56)	0.35 (0.10, 0.59); *p* = 0.01
*λ*_3_(×10^−3^mm^2^s^−1^)	1.56 (1.35, 1.77)	1.11 (1.02, 1.20)	0.45 (0.21, 0.69); *p* = 0.002
Haematoma			
T2 (ms)	88.7 (63.1, 114.4)	41.3 (38.3, 44.3)	47.4 (21.8, 73.1); *p* = 0.004
MD (×10^−3^mm^2^s^−1^)	1.81 (1.45, 2.18)	1.47 (1.36, 1.58)	0.35 (− 0.04, 0.73); *p* = 0.07
FA	0.21 (0.11, 0.32)	0.30 (0.27, 0.33)	0.09 (0.02, 0.19); *p* = 0.09
*λ*_1_(×10^−3^mm^2^s^−1^)	2.18 (1.82, 2.53)	1.95 (1.82, 2.09)	0.22 (− 0.12, 0.57); *p* = 0.16
*λ*_2_(×10^−3^mm^2^s^−1^)	1.76 (1.36, 2.15)	1.36 (1.17, 1.55)	0.39 (− 0.05, 0.84); *p* = 0.07
*λ*_3_(×10^−3^mm^2^s^−1^)	1.47 (1.02, 1.92)	1.09 (1.03, 1.14)	0.38 (− 0 09, 0.85); *p* = 0.08
Oedema			
T2 (ms)	49.1 (42.0, 56.1)	40.4 (42.0, 56.1)	8.7 (2.03, 15.4); *p* = 0.03
MD (×10^−3^mm^2^s^−1^)	1.71 (1.57, 1.86)	1.53 (1.42, 1.63)	0.18 (0.03, 0.34); *p* = 0.025
FA	0.22 (0.18, 0.27)	0.29 (0.24, 0.34)	0.07 (0.00, 0.13); *p* = 0.05
*λ*_1_(×10^−3^mm^2^s^−1^)	2.12 (1.99, 2.24)	2.02 (1.90, 2.14)	0.10 (− 0.05, 0.25); *p* = 0.18
*λ*_2_(×10^−3^mm^2^s^−1^)	1.64 (1.48, 1.81)	1.41 (1.28, 1.54)	0.24 (0.04, 0.43); *p* = 0.01
*λ*_3_(×10^−3^mm^2^s^−1^)	1.38 (1.20, 1.57)	1.16 (1.04, 1.27)	0.23 (0.04, 0.42); *p* = 0.02

### Visit 2 ROIs

**Fig. 3:**
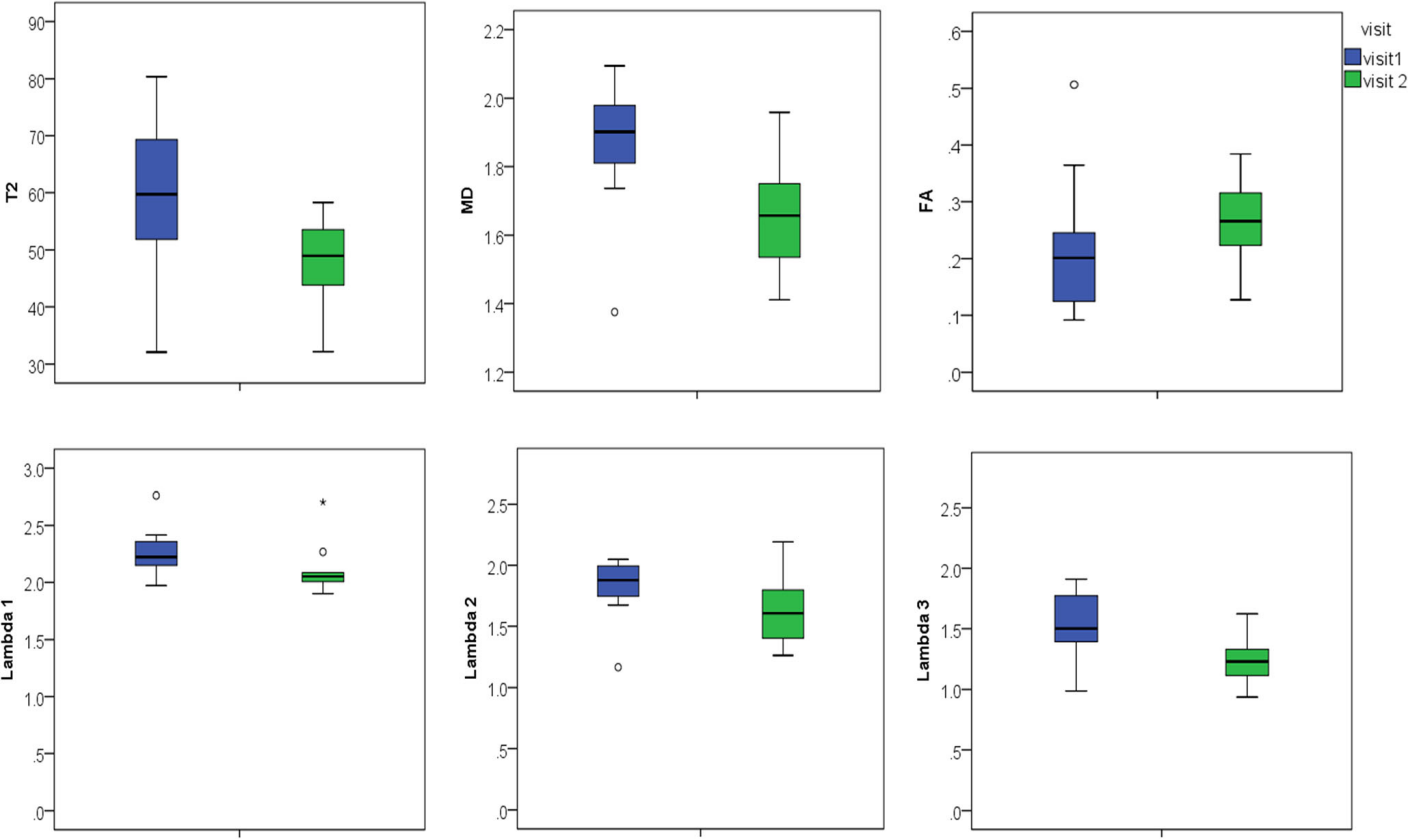
Boxplots for quantitative MR parameters obtained from ROIs drawn on visit 2 and propagated to visit 1. Measurements show tissue changes within the tear site only. Values for T2, MD and FA and eigenvalues in the tear site are presented

Considering the ROIs drawn on the visit 2 images within the tear site, there was still a significant reduction in T2, MD and *λ*_3_, and an increase in FA, with healing but differences in *λ*_2_ were no longer significant (Fig. 3, Table 3).

**Table 3:** Quantitative MR parameters obtained from ROIs drawn on visit 2 and propagated to visit 1. Measurements show tissue changes within the tear site only. Mean values for T2, MD and FA and eigenvalues for both visits and the difference between them are presented as mean (confidence interval); *p* value

Measurement	Visit 1 mean (CI)	Visit 2 mean (CI)	Difference mean (CI); *p* value
T2 (ms)	60.04 (49.73, 70.35)	47.34 (41.09, 53.59)	13.12 (4.82, 21.42); *p* = 0.01
MD (×10^−3^mm^2^s^−1^)	1.86 (1.72, 2.01)	1.65 (1.52, 1.77)	0.22 (0.04, 0.39); *p* = 0.02
FA	0.19 (0.13, 0.25)	0.26 (0.21, 0.32)	0.07 (0.01, 0.14); *p* = 0.03
*λ*_1_(×10^−3^mm^2^s^−1^)	2.23 (2.13, 2.32)	2.11 (1.94, 2.28)	0.12 (− 0.08, 0.31); *p* = 0.09
*λ*_2_(×10^−3^mm^2^s^−1^)	1.81 (1.62, 2.00)	1.63 (1.44, 1.83)	0.18 (− 0.08, 0.44); *p* = 0.16
*λ*_3_(×10^−3^mm^2^s^−1^)	1.55 (1.36, 1.74)	1.23 (1.09, 1.37)	0.32 (0.14, 0.50); *p* = 003

### Return to play time prediction study

**Fig. 4:**
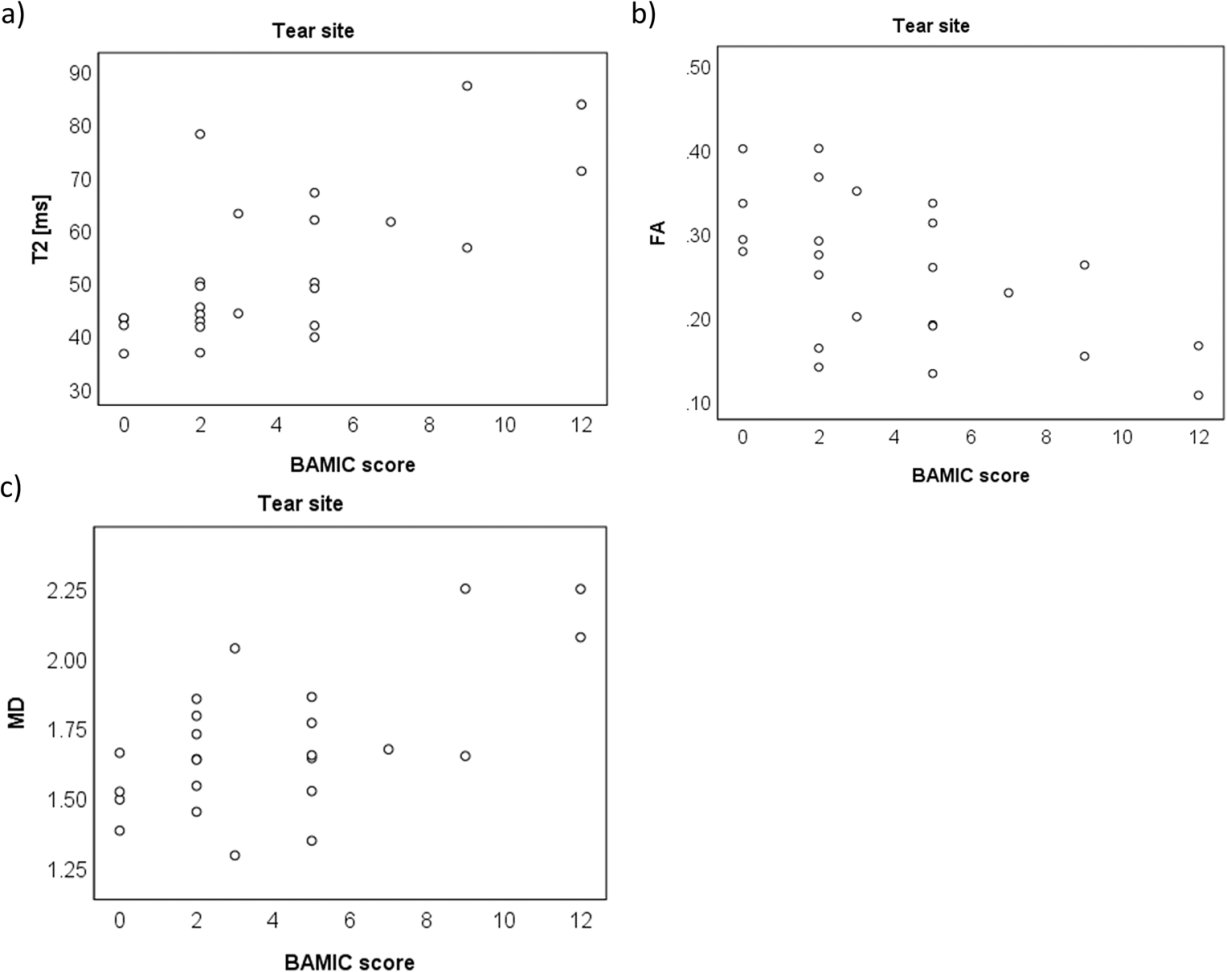
Scatter plots showing correlations between BAMIC scores and T2 (**a**), FA (**b**) and MD (**c**) in the tear site ROI

BAMIC and modified Peetrons scores correlated with T2, MD, FA, *λ*_2_ and *λ*_3_ values in the tear site and oedema ROIs (Fig. 4, Table 4). However, in the haematoma ROI, none of the measures correlated with radiologist’s scores.

**Table 4:** Spearman’s *R* values and *p* values for correlations between BAMIC and modified Peetrons scores compared with T2, MD and FA and eigenvalues in tear site, haematoma and oedema ROIs

Measurement	Score	Tear site	Haematoma	Oedema
T2 (ms)	BAMIC	*R* = 0.62; *p* < 0.01	*R* = 0.36; *p* = 0.18	*R* = 0.48; *p* = 0.01
	Peetrons	*R* = 0.59; *p* = 0.01	*R* = 0.51; *p* = − 0.05	*R* = 0.68; *p* < 0.01
MD (×10^−3^mm^2^s^−1^)	BAMIC	*R* = 0.49; *p* = 0.01	*R* = 0.27; *p* = 0.35	*R* = 0.52; *p* = 0.01
	Peetrons	*R* = 0.51; *p* = 0.01	*R* = 0.56; *p* = 0.04	*R* = 0.57; *p* = 0.01
FA	BAMIC	*R* = − 0.53; *p* < 0.01	*R* = − 0.32; *p* = 0.27	*R* = − 0.41; *p* = 0.04
	Peetrons	*R* = − 0.51; *p* = 0.01	*R* = − 0.19; *p* = 0.52	*R* = − 0.41; *p* = 0.04
*λ*_1_(×10^−3^mm^2^s^−1^)	BAMIC	*R* = 0.30 *p* = 0.15	*R* = 0.11; *p* = 0.70	*R* = 0.38; *p* = 0.06
	Peetrons	*R* = 0.29; *p* = 0.16	*R* = 0.56; *p* = 0.04	*R* = 0.36; *p* = 0.08
*λ*_2_(×10^−3^mm^2^s^−1^)	BAMIC	*R* = 0.56; *p* = < 0.01	*R* = 0.28; *p* = 0.33	*R* = 0.61; *p* = 0.001
	Peetrons	*R* = 0.56; *p* < 0.01	*R* = 0.52; *p* = 0.06	*R* = 0.56; *p* < 0.01
*λ*_3_(×10^−3^mm^2^s^−1^)	BAMIC	*R* = 0.61; *p* < 0.01	*R* = 0.41; *p* = 0.14	*R* = 0.54; *p* < 0.01
	Peetrons	*R* = 0.70; *p* < 0.01	*R* = 0.56; *p* = 0.04	*R* = 0.48; *p* = 0.02

**Fig. 5:**
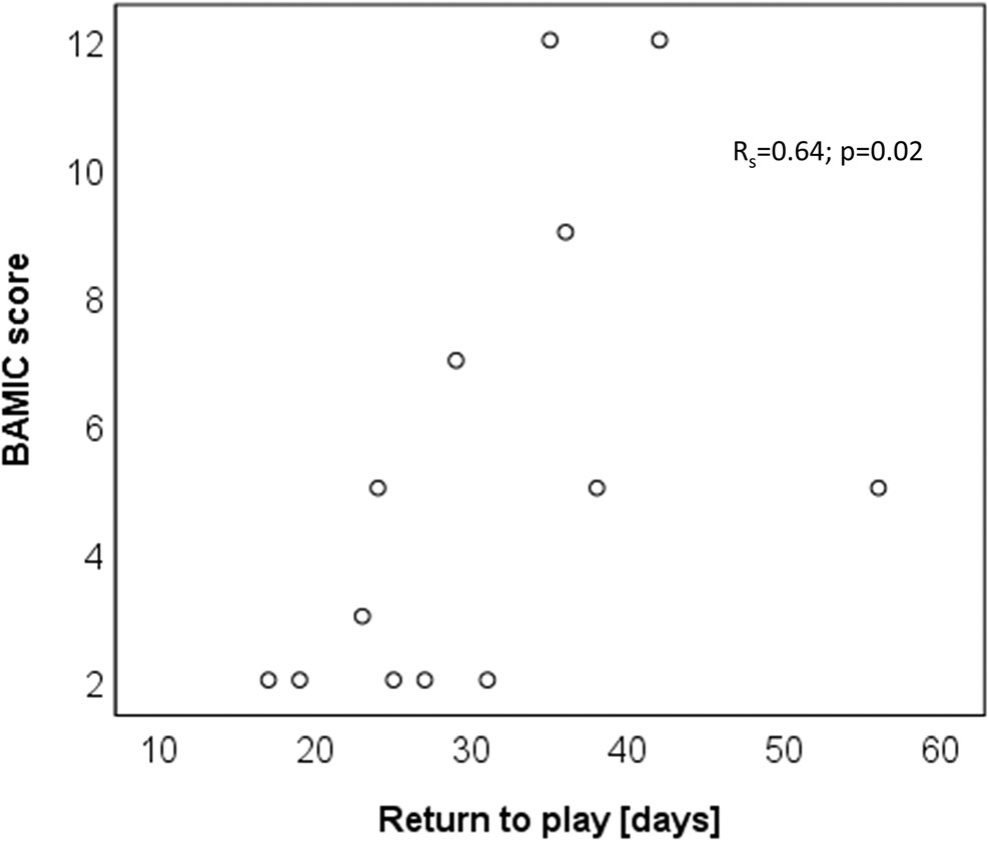
Correlation between radiologist’s BAMIC score and return to play time

**Fig. 6:**
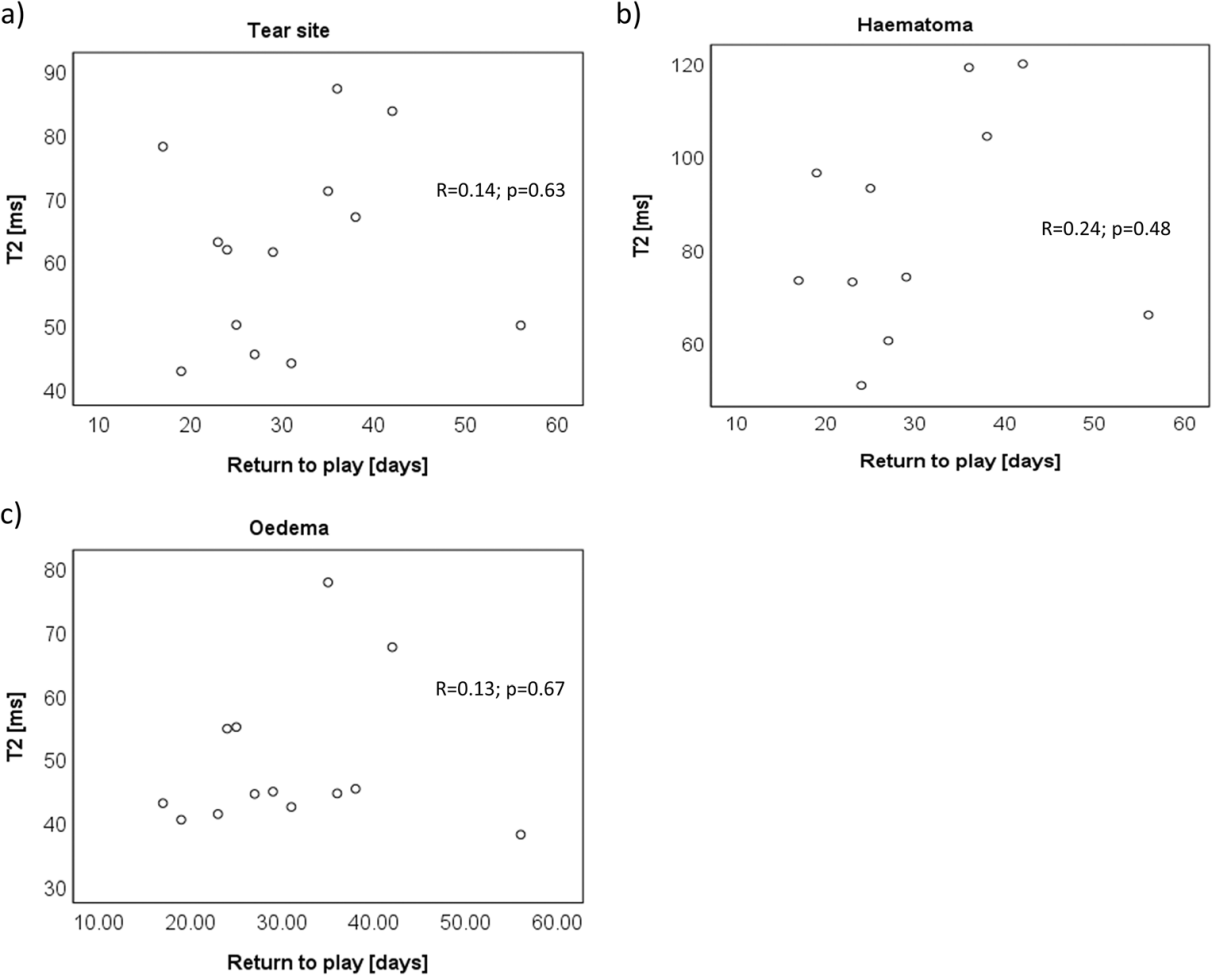
Scatter plots between T2 and return to play times in the tear site (**a**), haematoma (**b**) and oedema (**c**)

BAMIC scores showed a good correlation with return to play time (Fig. 5, *R*_s_ = 0.64; *p* = 0.02). Modified Peetrons score did not correlate with return to play time (*R*_s_ = 0.40; *p* = 0.18). None of the quantitative MRI measures correlated well with return to play time for any of the ROIs assessed (Fig. 6, Table 5).

**Table 5:** Pearson’s *R* values and *p* values for correlations between return to play time and visit 1 values for T2, MD and FA and eigenvalues in tear site, haematoma and oedema

Measurement	Tear site	Haematoma	Oedema
T2 (ms)	*R* = 0.14; *p* = 0.64	*R* = 0.24; *p* = 0.48	*R* = 0.13; *p* = 0.67
MD (×10^−3^mm^2^s^−1^)	*R* = 0.20; *p* = 0.51	*R* = 0.19; *p* = 0.58	*R* = 0.21; *p* = 0.49
FA	*R* = − 0.27; *p* = 0.38	*R* = − 0.28; *p* = 0.41	*R* = − 0.42; *p* = 0.15
*λ*_1_(×10^−3^mm^2^s^−1^)	*R* = 0.11; *p* = 0.71	*R* = − 0.04; *p*=0.92	*R* = 0.02; *p* = 0.95
*λ*_2_(×10^−3^mm^2^s^−1^)	*R* = 0.09; *p* = 0.77	*R* = 0.15; *p* = 0.67	*R* = 0.14; *p* = 0.64
*λ*_3_(×10^−3^mm^2^s^−1^)	*R* = 0.33; *p* = 0.28	*R* = 0.29; *p* = 0.39	*R* = 0.35; *p* = 0.24

## Discussion

This is the first study to use a longitudinal study design to assess quantitative measurements of T2 and diffusion in muscle tear. We have shown that T2 and DTI measurements are able to detect changes in muscle due to healing after muscle tear. Measurements showed differences when muscle tear size changes were included in the ROI (visit 1 ROI comparison; Table 2, Fig. 2) and when only changes within the tear site itself were considered (visit 2 ROI comparison; Table 3, Fig. 3). Although radiologist’s BAMIC scores correlated with return to play times, quantitative MRI values did not. Our results suggest that single-point ROI-based quantitative T2 and DTI measurements are not adequate predictors of return to play time. We would recommend radiologist’s BAMIC scores as having the most potential to predict return to play times post muscle tear in athletes.

The obtained values for T2 and MD agreed well with previous measurements in healthy muscle at 3 T [25, 29–31]. The differences seen using the visit 1 ROIs represent changes within the tear site itself and due to tear size reduction. These visit 1 measurements are of clinical importance because they are the only measurements that are available to a radiologist immediately after muscle tear. The observed reduction in T2 values and mean diffusivity is consistent with a reduction in fluid in response due to decreasing levels of oedema. The reduction in mean diffusivity is also consistent with fluid changes. The corresponding increase in fractional anisotropy could show restored fibre microstructure. As torn fibres are repaired, the inhomogeneity in fibre direction should decrease, increasing the FA. This is corroborated to some extent by the reduction in *λ*_2_ and *λ*_3_, which is consistent with reordering of the fibres due to healing but could also be affected by fluid reduction.

Analysis of the visit 2 ROIs allowed a separate analysis of changes within the tear site, excluding the effects of tear shrinking. Our results showed that T2 and MD measurements can detect reductions in fluid content within the tear site itself and not just due to reduction in the tear volume. Here, the increase in FA between visits could be due to fibre reordering within the tear.

Although BAMIC scores correlated well with return to play time, the modified Peetrons scale had an inadequate value range to be able to show a strong correlation, with 11/13 cases having a score of 2 at visit 1. This also explains why more Peetrons scores remained unchanged between visits than BAMIC. Given that BAMIC scores correlated with return to play time (supporting previous studies [2]) and with quantitative MRI, it is disappointing that quantitative MRI measurements did not independently correlate with return to play time. This is likely due to the limited scope of a single ROI-based measurement within the tear site compared with the radiologist’s score, which takes into account the tear size relative to the muscle belly, signal changes, position, and muscle retraction. Figure 6a shows a possible relationship, but the correlation is poor due to two outliers, with return to play times of 17 and 56 days. Excluding these outliers gives a correlation (*R* = 0.66; *p* = 0.02). On investigation, the 56-day point was also an outlier in the radiologist’s score plot (BAMIC of 2b) and the ROI placement seemed sensible. However, the 17-day point was not an outlier on BAMIC score (BAMIC = 1b) and had an exceptionally small tear site. The unusually high T2 value could be due to misplacement of the ROI and illustrates the potential limitations of ROI-based quantitative MRI as a tool in the muscle. Nevertheless, even when this point was excluded, the correlation between T2 and return to play time in the tear site remained low (*R* = 0.31; *p* = 0.33), so our conclusion remains that quantitative T2 and DTI values are not good predictors of return to play time.

There were limitations to our study. The sample size was small and the patient group was relatively heterogeneous. We did not correct for muscle changes due to age in our study. The relatively broad (19–35 years) age range of our participants might have decreased the ability to predict return to play time. There was a long delay (4 years) between acquisition of the data and submission of the paper for publication due to a number of factors including slow responses from the sports club over the 1-year follow-up data, the time taken to develop the post-processing software and staff changes at our institution. Imaging was performed within a week of injury, but a more stringent time window, such as 24–48 suggested by Ekstrand et al [6], may have given the MRI measurements more predictive power. A 3D registration approach might have reduced variation in slice position between visits. Although widely used, the use of a multi-echo sequence with a mono-exponential fit is susceptible to errors [20, 32]. Scoring was reported by only one radiologist and this could have been improved with a consensus reporting approach. Although blinded to BAMIC scores and tear size measurements, medical teams did receive a clinical report based on the first scan which could have biased return to play time estimates. Although it is currently the clinical reference measure, return to play time is a subjective measure and is therefore limited as a reference standard for degree of healing.

## Conclusions

T2 and DTI measurements in muscle can detect changes due to healing following muscle tear. T2 and MD values decrease, and FA increases, as the muscle heals. These trends are observed within the tear site itself and when changes due to shrinking of the tear volume are included in the ROI. In this study, radiologist’s BAMIC scores correlated with return to play times specified by the sports medicine team; however, modified Peetrons scores and quantitative MRI values did not. Our results suggest that single measurement, ROI-based quantitative T2 and DTI measurements are inferior predictors of return to play time compared with visual scoring.

## Abbreviations

BAMIC: British athletics muscle injury classification
DTI: Diffusion tensor imaging
FA: Fractional anisotropy
MD: Mean diffusivity
MRI: Magnetic resonance imaging
ROI: Region of interest
SPAIR: Spectral adiabatic inversion recovery
STEAM: Stimulated echo acquisition mode
